# Leveraging Machine Learning to Understand the Link Between School Climate and Youth Substance Use: a Focus on Cannabis and Alcohol Use

**DOI:** 10.1007/s11121-026-01887-2

**Published:** 2026-02-28

**Authors:** Ali Ünlü, Candra Skrzypek, Catherine P. Bradshaw

**Affiliations:** University of Virginia, Charlottesville, VA 22904 USA

**Keywords:** School climate, Alcohol, Cannabis, Marijuana, Machine learning, Risk factors

## Abstract

This paper focuses on school climate indicators, which have been previously linked with aspects of students’ well-being and school-related success, to explore how they relate to alcohol and cannabis use. We used machine learning (ML) approaches and leveraged data from a diverse sample of 69,513 students (45.4% White, 23.9% Black, 8.9% Latine) across 111 middle and high schools, with 12% (*n* = 7783) reporting cannabis use and 18.8% (*n* = 12,220) reporting alcohol use in the past 30 days. We focused on 154 items related to school climate, student attitudes and behaviors, and demographics. We employed a two-stage feature selection method, initially reducing the 154 features to 31, and subsequently to 20, for both alcohol and cannabis use. Alcohol and cannabis use shared 15 common features and 5 distinct features, though some variation occurred across these two outcome variables. We identified both unique and shared factors that best classified current users vs. non-users. Specifically, gender, sense of pride in the school, weapon carrying, and bullying others were unique indicators that best classified alcohol use. In contrast, difficulties overcoming challenges, problems controlling temper, and becoming angry easily were more strongly associated with cannabis use. Shared indicators associated with both substances included gang membership, skipping school, violent behavior, school–parent and school–student engagement, and gambling. The inclusion of diverse classification factors underscored ML’s ability to capture complex social and environmental factors that may be associated with substance use differently across student demographics. These features were tested in 12 classification models for both substances, achieving ROC-AUC scores up to 86% with fine-tuning of the best-performing models. The results highlight the utility of ML for examining complex, multidimensional indicators associated with substance use that complement traditional models.

**Supplementary Information:**

The online version contains supplementary material available at https://doi.org/10.1007/s11121-026-01887-2.

School climate is a well-established target of school improvement initiatives because of its association with a number of youth outcomes, including academic achievement, reduced engagement in delinquent and problem behavior (Bradshaw et al., 2021). For example, positive school climates may be a protective factor against risky student behaviors, such as substance use (Mayberry et al., 2009; Michael et al., 2015). While theory-driven statistical models provide a strong foundation for understanding these associations, their ability to simultaneously incorporate the multidimensional nature of school climate and related correlates is often constrained by assumptions and linearity. By contrast, machine learning (ML) approaches offer a data-driven perspective that can capture complex patterns across multiple dimensions (Casari & Zheng, 2018), enabling a more nuanced understanding of school climate and its differential impact on youth behavior across diverse populations.

This study used ML to examine the association between school climate and student substance use, focusing specifically on alcohol and cannabis use. Leveraging a large and diverse sample of nearly 70,000 middle and high school students, we applied a multi-stage ML analytic strategy, including feature selection, model training, and hyperparameter tuning, to identify complex, multidimensional indicators that help classify current users from non-users. This data-driven approach enables the detection of nonlinear patterns and high-dimensional interactions, offering insights that may inform policy and prevention programming in educational settings. Specifically, the first aim used ML to identify the most informative set of indicators for classifying alcohol and cannabis users. We focused on 154 youth self-reported indicators of school climate and related variables (e.g., internalizing and externalizing problems, future aspirations, student wellness, stress, and student demographics). We were interested in whether these associations with adolescents’ substance use varied as a function of youth gender (Blair & Luo, 2024). Therefore, our second aim examined whether the school climate and related indicators associated with the classification of alcohol and cannabis users identified in Aim 1 varied by gender. Together, these analyses highlight the utility of ML as an analytic tool for detecting nuanced risk factors, which in turn may support more equitable and targeted school-based interventions to reduce adolescents’ use of these two health-compromising substances.

## Substance Use in Adolescence

Adolescence is a critical developmental stage characterized by rapid physical, cognitive, and psychosocial development. During this stage, adolescents often display increased impulsivity and engage in risky behaviors, including substance use (Steinfeld & Torregrossa, 2023). Substance use is typically initiated during adolescence, with alcohol and cannabis being two of the most used substances (Gray & Squeglia, 2018). In addition to nicotine, these substances are readily available to adolescents, are often perceived as being low-risk, and commonly used in social settings (Steinfeld & Torregrossa, 2023). Overall, the percentage of students who use alcohol or cannabis has decreased over the past decade (CDC, 2023), including significant declines during the COVID-19 pandemic (Miech et al., 2024). In fact, 2024 saw record high levels of drug abstention (i.e., no past 30-day use of alcohol, marijuana, or nicotine) among students (Miech et al., 2024). Despite these promising trends, adolescent use of substances is associated with a myriad of risks (Gray & Squeglia, 2018; Steinfeld & Torregrossa, 2023). Data from the Youth Risk Behavior Surveillance System (YRBS) suggested that in 2023, the percentage of high schoolers who drank alcohol in the past 30 days was 22% while the percentage of those who reported using cannabis was slightly lower at 17%. These rates were higher for females, suggesting females were more likely than males to both drink alcohol (24% compared to 20% for males) and use cannabis (19% compared to 15% for male students). Alcohol and cannabis use also remain less common among middle school students compared to those in high school (Miech et al., 2024).

Adolescent substance use is influenced by the various ecological contexts (e.g., peers, family, school, community) where development occurs. Each of these contexts can potentially provide protective and risk factors for substance use and operate individually or in combination. While research indicates that peers and parents tend to have the strongest and most direct influence on adolescent substance use, the school context also contributes to adolescent substance use indirectly (Trucco, 2020) and might be an especially important protective factor for older adolescents (Cleveland et al., 2008). It has been noted that school climate may also be a particularly salient protective factor for youth for substance use (Trucco, 2020).

Adolescent substance use has been linked with numerous negative consequences, including poor school performance, reduced cognitive functioning, the development of neuropsychiatric disorders, and substance use disorders in adulthood (Gray & Squeglia, 2018; Steinfeld & Torregrossa, 2023). Several factors at the individual, familial, and social levels increase the likelihood of adolescent substance use initiation and problematic use. Prevention efforts for adolescent substance use have focused on reducing modifiable risk factors and promoting protective factors across domains. One area of growing interest is the association between substance use and school-related factors, including the school climate.

### School Climate and Adolescent Substance Use

The term “school climate” has been defined as a multidimensional construct that generally characterizes the perceptions and norms of the school environment. The U.S. Department of Education (ED) conceptualized school climate in terms of engagement (e.g., strong relationships between teachers and students, student connectedness), safety (e.g., emotional and physical safety), and the environment (e.g., the disciplinary context and the physical environment). A positive school climate has been associated with a number of favorable academic and behavioral outcomes for students, including improved attendance rates, test scores, graduation rates, reduced discipline problems, and improved adolescent mental health (Bradshaw et al., 2021; Suldo et al., 2012; Thapa et al., 2013). Further, positive school climate may promote healthy student behaviors and be a protective factor against risky student behaviors, such as substance use (Mayberry et al., 2009; Michael et al., 2015). Schools characterized by authoritative school climates (i.e., having clear and consistent, but fair discipline and supportive teacher-student relationships) are associated with lower levels of alcohol and cannabis use among students (Cornell & Huang, 2016). Supportive teacher-student relationships may be particularly important; another study of over 900 low-income minority adolescents found that teacher support was associated with lower odds of 30-day use of cannabis. This association was mediated by behavioral self-concept, suggesting that teachers play an important role in how adolescents develop their sense of self and avoid risky behaviors (Dudovitz et al., 2017).

School climate interventions may be especially important during the middle grades (Daily et al., 2020), as substance use typically increases with age (Miech et al., 2024), whereas perceptions of school climate often decline from middle to high school (Waasdorp et al., 2020). Another recent study of middle-grade (6–8) youth classified schools as either having high or low proportions of students with a high probability of early polysubstance use (i.e., heavy drinking, cannabis, and tobacco). Schools with higher proportions of students with higher probabilities of polysubstance use had lower average school climates, belonging (e.g., feeling close to people at school), and safety scores than schools with lower proportions (Halladay et al., 2024). In this study, the effects of school environmental factors did not differ between males and females, suggesting that differences may not arise until later adolescence (Halladay et al., 2024). Other studies with older adolescents have found that school belonging might have larger protective effects for females against substance use and mental health problems (Halladay et al., 2022).

### Overview of the Current Study

Taken together, the extant research suggests that a positive school climate may be related to adolescent alcohol and cannabis use. Still, much of the literature examining this association has focused on specific school climate domains or factors (e.g., student–teacher relationships) instead of simultaneously exploring specific indicators (Ko et al., 2023). Building on prior research linking a positive school climate with less alcohol and cannabis use (Mayberry et al., 2009), the current study sought to address gaps regarding the specific aspects of this multidimensional construct that might be linked with specific forms of substance use and explore whether those associations might vary by gender. We focused on 154 self-reported indicators of school climate, as well as other relevant constructs including internalizing and externalizing problems, future aspirations, student wellness, stress, and student demographics (Bradshaw et al., 2014). We used ML to identify which specific items were most strongly associated with alcohol and cannabis use. ML provides a data-driven approach that can uncover complex, nonlinear relationships and interactions among numerous variables, enabling more precise identification of specific school climate components linked to adolescent substance use.

Specifically, the first aim was to identify the most salient items and assess their contribution to classifying current substance use status among adolescents. In the current study, ML models were used to distinguish or “classify” substance users from non-users based on concurrently measured features, including for previously unseen cases (Bzdok et al., 2018). However, given the study’s cross-sectional design, the classification does not imply any temporal or causal inference regarding future substance use. Our second aim was to explore variation in factors by gender, as prior research suggests potential differential impacts of school climate on youth outcomes by gender.

## Method

### Data

Data come from youth self-report on the Maryland Safe and Supportive School (MDS3) Climate Survey (Bradshaw et al., 2014) during the 2014–2015 school year, comprising responses from 69,513 students nested within 3266 classrooms across 114 schools. Of these participants, 47% (*n* = 32,513) identified as male and 46% (*n* = 32,127) as female; 7% (*n* = 4,873) did not disclose their gender. In terms of racial and ethnic composition, the majority of students were White (45.4%, *n* = 31,556), followed by Black (23.9%, *n* = 16,628) and Hispanic/Latino (8.9%, *n* = 6,230). The remaining 21.8% (*n* = 15,099) comprised students who identified as Native American/American Indian, Asian/Pacific Islander, Native Hawaiian or Other Pacific Islander, those categorized as “Other,” or those who did not report their race or ethnicity. Of students reporting their grade level, 46% (*n* = 29,720) were in grades 6–8, while 54% (*n* = 34,950) were in grades 9–12 (also see for more details Lindstrom Johnson et al., 2019).

The Maryland Safe and Supportive School (MDS3) Climate Survey (Bradshaw et al., 2014) was developed to assess the ED’s three-factor model of school climate, which includes safety, engagement, and environment domains. While the survey includes validated subscales for each domain, the current study utilized 154 individual self-reported items drawn from across these domains, rather than the aggregated subscale scores. These items reflect a broad range of school climate constructs, as well as other areas, such as internalizing and externalizing problems, future aspirations, student wellness, stress, and demographic characteristics (see Bradshaw et al., 2014). A full list of items included in the analysis is provided in Supplemental Table 1. Prior studies of the MDS3 Survey have demonstrated strong psychometric properties and measurement invariance across gender, race, and grade level (see Bradshaw et al., 2014; Lindstrom Johnson et al., 2019; Waasdorp et al., 2020). The anonymous survey was completed online by the students, using a waiver of active parental consent and youth assent process, in partnership with the Maryland State Department of Education (MSDE). The non-identifiable data were obtained from MSDE for analysis. All procedures were approved by the researchers’ Institutional Review Board.

**Table 1:** Top performing variables

Variable label		Status
Have you ever belonged to a gang?		Common
During the last month, how many days of school have you missed because you skipped or “cut”? (i.e., 0 days to 6 or more days, on a 4-point scale)		Common
I have threatened to hit or hurt someone		Common
I do all my schoolwork		Common
My teachers encourage me to work hard in my classes		Common
It is okay to hit someone if they hit me first		Common
Get into a lot of trouble		Common
If I do something bad at school, my parent(s) or guardian(s) hears about it		Common
When I do something good at school, my parent(s) or guardian(s) usually hears about it		Common
Have you ever gambled? (response *never*)		Common
How old are you? (i.e.,* age*)		Common
My teachers believe that I can do well in school		Common
I do things without thinking		Common
My teachers make me feel good about myself		Common
The principal at my school cares about us students		Common
I believe I can do well in school		Alcohol
During the past 30 days, how often did you carry a weapon, such as a knife or gun, on school property? (i.e., 0 days to 6 or more days, on a 4-point scale)		Alcohol
Gender		Alcohol
In the past 30 days, how often have you bullied someone else? (i.e., 4-point scale: “Not at all” to “Once a week or more”)		Alcohol
At this school, students and staff feel pride in this school		Alcohol
Felt that difficulties were piling up so high that you could not overcome them		Cannabis
I have trouble controlling my temper		Cannabis
At this school, my teachers care about me		Cannabis
I get mad easily		Cannabis
I like this school		Cannabis

The age variable is continuous, while *Gender* is binary. All other items are ordinal. Higher values indicate greater risk or agreement with the statement

### Analyses

**Fig. 1:**
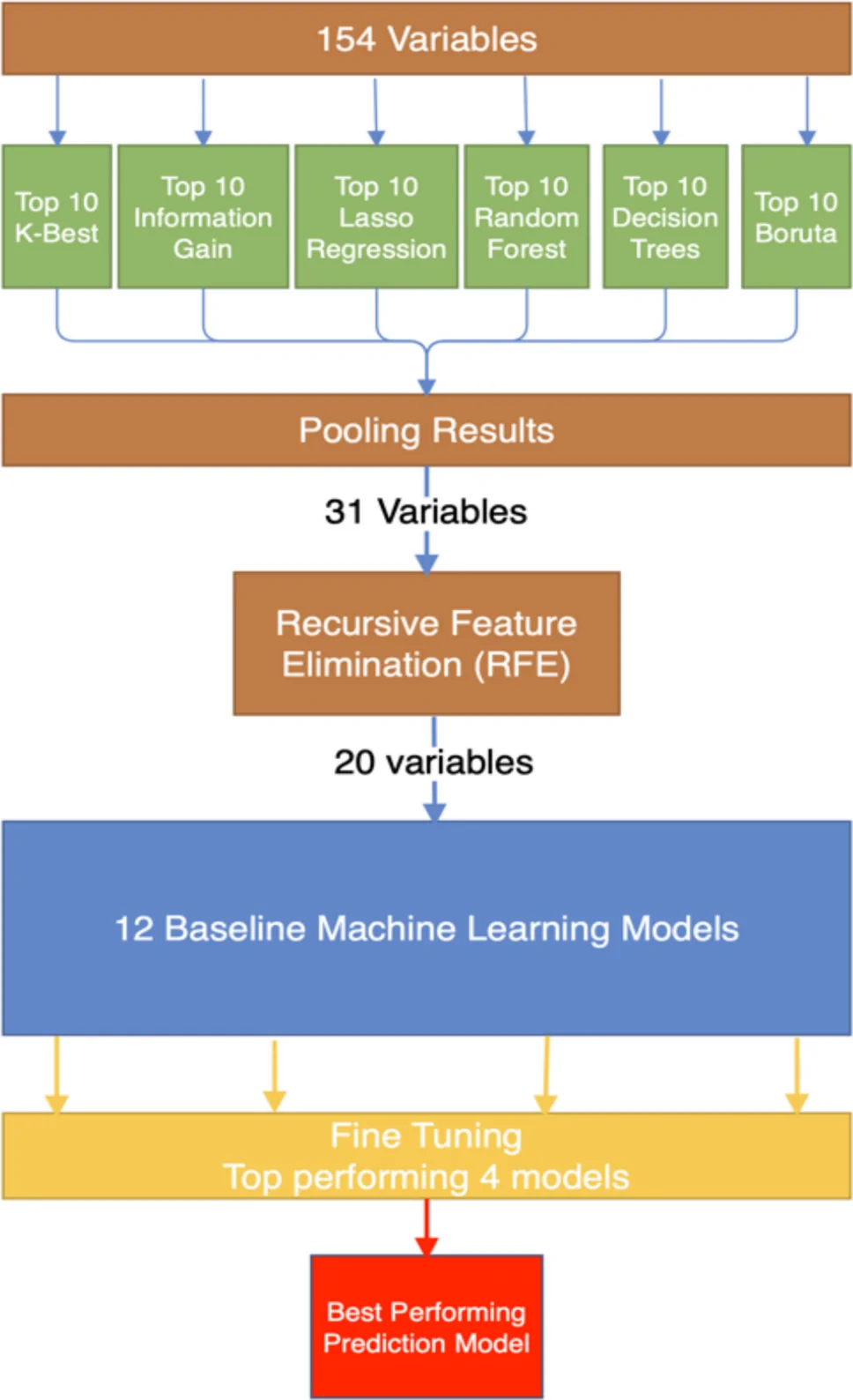
Prediction model building process

Our analytic strategy began with data preprocessing, followed by a two-stage feature selection approach (Fig. 1). After identifying the top 20 features associated with per outcome, we conducted classification modeling by testing 12 different ML algorithms and applying hyperparameter tuning to evaluate the performance of the selected features (Fig. 1). Once the best-performing model was identified, we finalized the classification model. In the final stage, we analyzed variations in factors across gender groups.

Data preprocessing began with case-wise deletion of observations with more than 50% missing data, resulting in a final sample of 60,488 cases. Binary (e.g., “Have you ever belonged to a gang?” (yes/no)) and continuous variables (e.g., age) were retained in their original formats, whereas most variables, measured on Likert scales, were evaluated for sparsity and skewness. We dichotomized only a highly sparse or skewed Likert-scale variable to improve interpretability and model performance. Missing data patterns were examined, revealing item-level missing rates ranging from 0 to 9%. Missing values were imputed using the k-nearest neighbors (KNN) algorithm, a widely used ML method designed to preserve local data structure during imputation (Pedregosa et al., 2011). To address potential multicollinearity, we conducted pairwise Pearson correlation analyses. Only four items exhibited moderate correlations between 0.70 and 0.75, while all remaining correlations fell below 0.70. Given these results, no items were excluded due to multicollinearity. A stratified split method was employed to divide the data into three subsets—training, validation, and test—using respective ratios of 70%, 15%, and 15% for all models described below.

### Variable Selection

All 154 individual-level items from the MDS3 Survey (Bradshaw et al., 2014) were analyzed to identify key classifiers of cannabis and alcohol use among students. To maintain a theoretical focus on contextual and environmental influences, we deliberately excluded items that directly assessed students’ personal substance use behaviors, such as self-reported cigarette use, illicit drug use, and non-medical prescription drug use, as well as items related to perceived health risks and substance accessibility. While we acknowledge that including these items could improve predictive performance and may rank highly in SHAP importance scores, their inclusion would overshadow the specific contribution of school climate factors—our primary focus. This decision was therefore theory-driven rather than classification-driven, which aligns with our aim to inform structural and relational interventions within schools.

The outcome variables, cannabis and alcohol use, were initially measured on a 7-point scale reflecting usage frequency over the past 30 days (Bradshaw et al., 2014) and were based on similarly worded YRBS indicators. Given the relatively low prevalence of use within the student population, these variables were recoded into binary formats. Specifically, 12.8% (*n* = 7758) of students reported cannabis use at least once in the past 30 days, while 20% (*n* = 12,176) reported alcohol use within the same timeframe.

### Feature Selection

Given the dimensionality of the dataset and the number of items included in the classification model, we implemented a two-stage feature selection process to isolate the most relevant classification factors, reduce model complexity, and minimize the risk of overfitting. By focusing on items that contribute the most to classification accuracy, we aimed to improve both model interpretability and performance (Kuhn & Johnson, 2020; Raschka et al., 2022). The initial stage employed filter-based and univariate methods, including variance thresholding, K-best, and information gain techniques. Subsequently, we utilized embedded methods, such as Lasso regression, Random Forest, and Decision Trees. Additionally, we applied the Boruta feature selection method in conjunction with LightGBM, XGBoost, and Random Forest classifiers. Throughout these analyses, the data were normalized using RobustScaler, which centers variables on the median and scales them according to the interquartile range. This approach is particularly well-suited for Likert-type items, which often display skewed distributions (Pedregosa et al., 2011). All models were evaluated using tenfold cross-validation. Class weights were incorporated, where supported, to address imbalanced data distributions (Casari & Zheng, 2018; Pedregosa et al., 2011). Feature selection procedures were conducted independently for cannabis and alcohol use. For each outcome variable, the top 10 features identified by each algorithm were pooled, resulting in 31 distinct classifying indicators.

While the first stage reduced the feature set from 154 to 31 items, Recursive Feature Elimination (RFE), an advanced feature selection method, was then used to further reduce the set from 31 to 20 items. This decision was based on classification performance, as RFE results indicated that feature sets containing between 15 and 20 items yielded optimal and stable performance across models (Fig. 1). To ensure robust feature selection, RFE was applied using multiple algorithms, including Decision Trees, Gradient Boosting Classifier (GBC), Support Vector Machines (SVM), Random Forest, Perceptron, XGBoost, and Extra Trees algorithms. To address class imbalance (i.e., most respondents did not report cannabis or alcohol use), we adopted a SMOTE-Tomek approach while retaining tenfold cross-validation for robustness and generalizability. After testing other resampling strategies for Tomek Link, we determined that “not majority” resampling strategy yielded the most favorable results. The Tomek link is a hybrid technique that removes instances from the majority class, effectively shifting the decision boundary and prioritizing the minority group. By combining SMOTE for oversampling and Tomek link for undersampling, this method effectively mitigates data imbalance (Casari & Zheng, 2018; Pedregosa et al., 2011; Unlu et al., 2025; Wang et al., 2019).

### Classification Models

We employed a consistent analytical framework aligned with prior stages, including data scaling, tenfold cross-validation, and addressing class imbalance using SMOTE-Tomek. ML models typically perform best when the outcome classes are balanced; however, class imbalance is a common challenge in behavioral science research (Han et al., 2020; Wadekar, 2020). In our case, the minority class—students reporting substance use—was underrepresented, which can lead to models biased toward the majority class (i.e., non-users) due to insufficient learning from rare events. To address this issue and reduce algorithmic bias, we applied SMOTE to augment the training dataset. Importantly, SMOTE was applied only to the training set and not to the validation or test sets, thereby preserving the integrity of model evaluation. To maintain a conservative approach, the minority class was oversampled by 40% during training. We also replicated the analyses without applying SMOTE during training as a robustness check. The results show that SMOTE preserves the underlying data structure, does not distort variable importance, and enhances classification performance (Fernández et al., 2018; Pedregosa et al., 2011; Raschka et al., 2022; Unlu et al., 2025).

An initial evaluation was conducted using 12 classification algorithms with default parameters to identify those best suited for the classification task. Given the imbalanced nature of the data, we prioritized precision-based metrics to reduce the likelihood of false positives. This approach helps avoid incorrectly labeling non-users as users, which is important in contexts where such misclassification could lead to undue concern or intervention. Specifically, we used average precision during model tuning, which provides a more informative assessment of model performance under class imbalance than overall accuracy. Additionally, class weighting techniques were applied, where supported, to ensure that the models gave adequate attention to the minority class. For example, in models such as Random Forest and XGBoost, weights were adjusted so that misclassifying substance users would carry more penalty than misclassifying non-users. While ROC-AUC was retained as the primary evaluation metric for comparability across models, these adjustments were essential to ensure fair learning from both majority and minority classes.

Previous research indicates that ML models yield similar or superior results compared to deep learning models for this type of task (Mak et al., 2019; Parekh & Fahim, 2021; Unlu & Subasi, 2025; Unlu et al., 2023). Therefore, we limited our testing of deep learning models to artificial neural networks (ANNs). To ensure robust model comparison, we evaluated a diverse set of algorithms representing different modeling paradigms, including linear and probabilistic models (Logistic Regression, Linear Discriminant Analysis, Naïve Bayes), instance-based and tree-based methods (k-Nearest Neighbors, Decision Trees), ensemble approaches (AdaBoost, Gradient Boosting Machines, Random Forest, Extra Trees, Bagging, XGBoost), and neural networks (ANNs). This diversity allowed us to assess classification performance across models with varying assumptions, complexity, and capacity to capture nonlinear relationships. The top four performing models were subjected to further optimization through hyperparameter tuning.

Hyperparameter tuning refers to the process of optimizing the configuration settings that govern how an ML algorithm learns from data (e.g., tree depth in decision trees, learning rate in boosting algorithms). These parameters are not learned from the data directly but can significantly affect model performance. For this study, we utilized RandomizedSearchCV from the Scikit-Learn package (Pedregosa et al., 2011) to search for optimal values across predefined grids for each algorithm. Model performance was evaluated using multiple metrics, including precision, recall, accuracy, F1 score, Cohen’s Kappa, Matthews Correlation Coefficient (MCC), and ROC-AUC (Raschka et al., 2022). Consistent with prior literature, ROC-AUC values closer to 1.0 indicate stronger discriminative ability, while F1 scores provide a balance between precision and recall in the presence of class imbalance (Raschka et al., 2022).

To identify indicators that differentiate alcohol and cannabis users, we applied SHAP (SHapley Additive exPlanations) to the data without SMOTE augmentation. The SHAP is an ML interpretability framework that quantifies the contribution of individual features to model classification (Nohara et al., 2019). SHAP values quantified the contribution of each feature to the classification probability of a specific outcome (Lundberg & Lee, 2017). We used the XGBoost classification model to analyze items associated with alcohol and cannabis users (class = 1) and non-users (class = 0), as it was identified as the best-performing algorithm in our analysis, as described below. Positive SHAP values indicate an increased likelihood of substance use, whereas negative values suggest a reduced likelihood (Lundberg et al., 2020). To investigate gender-specific differences in classification, we computed SHAP values separately for male and female subsets of the data. This facilitated a comparative analysis of feature importance across genders, highlighting indicators with differential classification strength for males and females.

## Results

### Top Features

The top 20 features (Table 1) identified for cannabis and alcohol use classification models revealed both commonalities and distinct patterns. Fifteen features were shared across both cannabis and alcohol use. Common features included items such as “I have threatened to hit or hurt someone” and “I do things without thinking.” Five distinct indicators were also identified for both alcohol use and cannabis use.

### Prediction Models

After evaluating 12 ML algorithms, we selected the four top-performing models for fine-tuning (Fig. 1). XGBoost demonstrated the best overall performance across all metrics when SMOTE was applied, while Random Forest outperformed other models in the absence of SMOTE for two outcomes. For cannabis use, the model achieved an ROC-AUC of 0.86, with precision and F1 scores of 0.87 and recall values of 0.86, which indicates strong overall performance (Table 2). Similar results were observed in models trained without SMOTE (Supplemental Table 3). In addition to standard performance metrics, we also evaluated Cohen’s Kappa (0.42) and Matthews Correlation Coefficient (MCC) (0.42) to assess model agreement and classification quality (Table 2). For alcohol use, the model achieved an ROC-AUC of 0.84, with a precision of 0.82, a recall of 0.79, and an F1 score of 0.80. Cohen’s Kappa and MCC were 0.42 and 0.43, respectively (Table 2). While models trained without SMOTE produced similar results, applying SMOTE notably improved recall, thereby enhancing the model’s ability to identify alcohol users in this imbalanced classification task (Supplemental Table 3).

**Table 2:** Model results with SMOTE

	Models	Accuracy	Precision	Recall	F1 score	Cohen’s Kappa	MCC	ROC-AUC
Cannabis	XGBoost	0.86	0.87	0.86	0.87	0.42	0.42	0.86
Alcohol	XGBoost	0.79	0.82	0.79	0.80	0.42	0.43	0.84

*MCC* Matthews Correlation Coefficient, *ROC-AUC* Receiver Operating Characteristic-Area Under the Curve

Although Cohen’s Kappa and MCC metrics are known to be sensitive to class imbalance and may be less stable in binary classification tasks (Delgado & Tibau, 2019; Zhu, 2020), we included them to support model comparison, particularly given the marginal differences in ROC-AUC across models. Following established interpretations, a Kappa value above 0.40 indicates moderate agreement (Landis & Koch, 1977), while MCC values above 0.40 are often considered indicative of informative performance in imbalanced classification scenarios, despite the absence of universal thresholds (Boughorbel et al., 2017; Chicco & Jurman, 2020).

The application of SMOTE did not change ROC-AUC scores in any condition, but improved recall performance in alcohol slightly. Given the inherent class imbalance in behavioral data, we prioritized model results trained with SMOTE. This approach improves the model’s ability to learn from the minority class without introducing bias into validation or test evaluations. While performance metrics were similar in non-SMOTE models (Supplemental Table 3), the SMOTE-trained models offered more balanced treatment of rare class predictions and are therefore presented as the primary findings. Additionally, our analysis revealed that school climate items demonstrated greater classification power for cannabis use compared to alcohol use. This finding remained robust across all conditions.

### Feature Importance on Substance Use

**Fig. 2:**
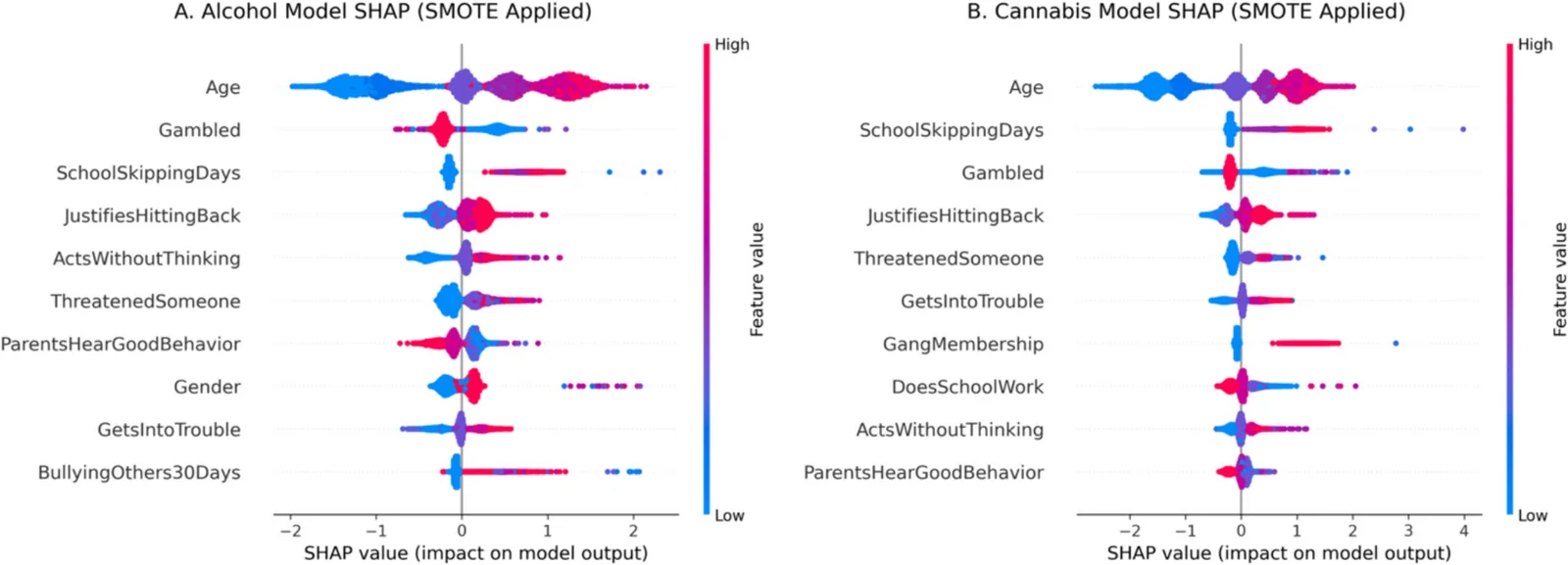
Feature importance for cannabis and alcohol ise. Note: SHAP summary plots for alcohol use (**A**) and cannabis use (**B**) highlight the feature importance and direction of influence on the model predictions (class 1 = substance use). Each point represents an individual data instance, with color indicating the feature value (red = high, blue = low). See specific items corresponding to the variable labels on Table 1

Figure 2 presents the direction and strength of each feature’s contribution to substance use classification. SHAP values show how each feature influences the model’s classification: positive values increase the probability of being classified as a user, while negative values indicate being a non-user. To ensure that applying SMOTE during model training did not substantially alter the data structure or feature importance, we present SHAP analysis results trained without SMOTE (Supplemental Fig. 1). For alcohol use, the top 10 predictors remained identical across both conditions, with only minor differences in their rankings. In the case of cannabis use, the top 10 list differed by a single feature: *Parents Hear Good Behavior* was replaced by *Overwhelmed By Difficulties* when SMOTE was applied (Fig. 2, Panel B). This substitution occurred at the 10th position, indicating only a marginal change among the lower-ranked features.

Among the common indicators, *Age* emerged as the most significant feature for both cannabis and alcohol use, with older individuals being more likely to be classified as substance users. Similarly, *School Skipping Days* ranked high for both substances, indicating that disengagement from school is closely associated with substance use behaviors. *Gambling* history was another shared indicator, suggesting that risky behaviors often co-occur across different domains. Three additional items, *Justifies Hitting Back*, *Threatened Someone,* and *Acts Without Thinking,* indicate that externalizing problems are associated with substance use. *Parents Hear Good Behavior* appeared in both models as a lower-ranked protective factor, potentially reflecting the influence of positive parental engagement (Fig. 2).

Despite these shared predictors, distinct patterns emerged for each substance. For cannabis use, features such as *Gang Membership* and *Does School Work* were stronger contributors, suggesting that school attachment and behavioral regulation are particularly relevant in this context. In contrast, alcohol use was more strongly associated with *Bullying Others in the last 30 Days* and *Gender,* reflecting the influence of interpersonal risk and demographic factors. Furthermore, *Gender* showed a more pronounced association with alcohol use patterns compared to cannabis (Fig. 2).

### Gender Differences in Alcohol Use

**Fig. 3:**
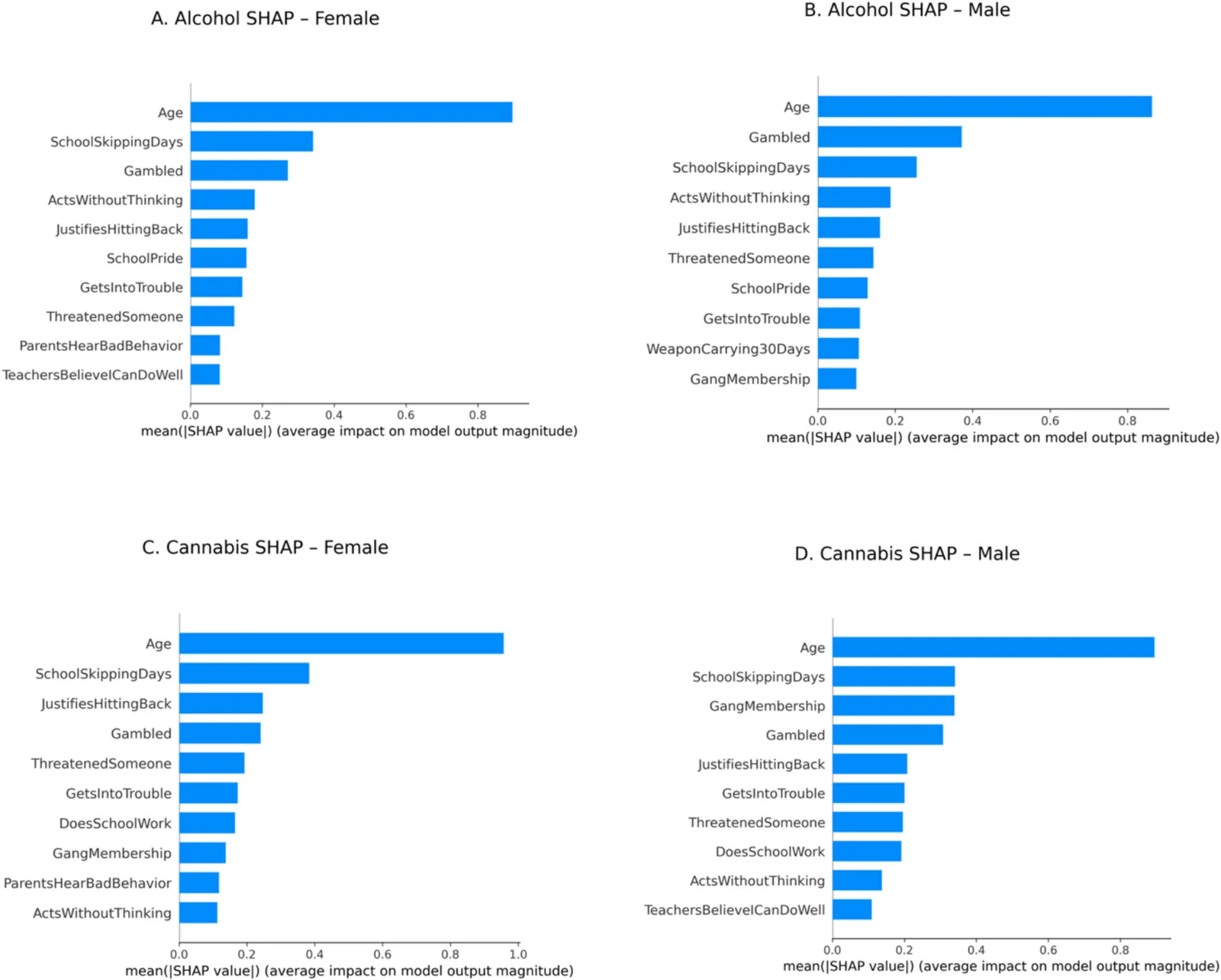
Feature importance across gender

**Fig. 4:**
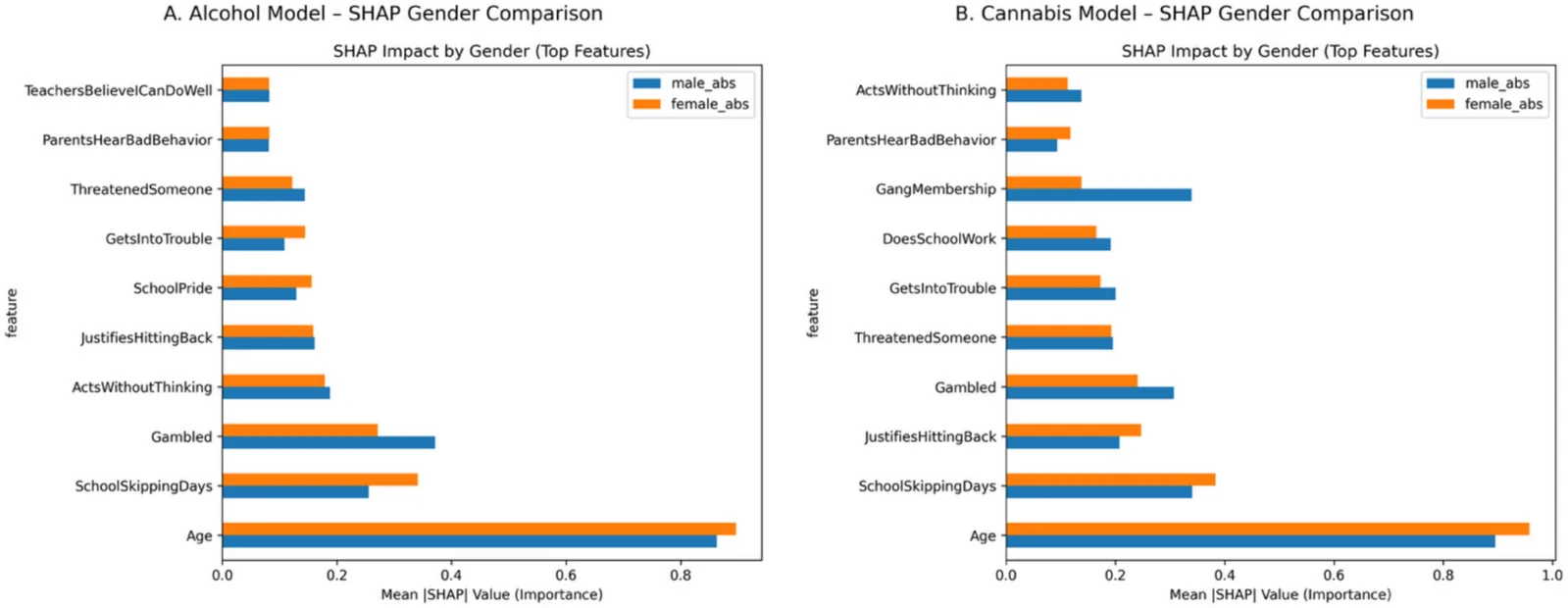
Feature importance comparison use across gender

As shown in Figs. 3A and 4A (see Supplemental Fig. 3 for details), *Age* clearly stands out as the strongest risk factor for both genders, with a slightly stronger impact (i.e., elevated risk) among female respondents. The next most influential features include *School Skipping Days* and *Gambled*, which appear consistently across both genders. While school skipping has a stronger effect for females, gambling history shows a greater effect among males.

*Threatening or Hurting Someone* is also a key risk factor for both genders, but more prominent for females in alcohol use, and slightly stronger for males in relation to cannabis use. *Act Without Thinking* (stronger for males), *Justifies Hitting Back* (stronger for females), and *Get into Trouble* (stronger for females in alcohol, but stronger for males in cannabis) also emerged as shared behavioral indicators across genders. In contrast, *Weapon Carrying* and *Gang Membership* appeared only in the top 10 features for males, which suggested their specific relevance in classifying alcohol use among male students (Fig. 3B).

On the protective side, “If I do something bad at school, my parents hear about it,” showed a stronger effect among females and only appeared in the female top 10 feature list (Fig. 3), suggesting that parental monitoring may play a more protective role for girls. Similarly, “Teachers believe I can do well in school” was also among the top 10 features for females. However, *School Pride* emerged as a key protective factor for both genders*.* In addition, several of the school-engagement indicators, such as doing homework, teacher encouragement to work hard in classes, and the teacher making the student feel good also appeared to serve as buffers against alcohol risk for both genders, but were slightly stronger for females (Supplemental Fig. 2). This suggests that school engagement and teacher-student relationships may have a particularly important role for girls in buffering alcohol-related risks.

### Gender Differences in Cannabis Use

In these SHAP results (Figs. 3C/D, 4B; see also Supplemental Fig. 3 for details), *Age* emerged as the strongest classifying indicator across genders, a pattern similar to alcohol, with a slightly stronger effect for females. A school-related item, *Skipping School Days*, ranked second for both genders, highlighting the importance of school attendance in differentiating cannabis users. Impulsivity and anger-related items (e.g., *Threatening or Hurting Someone* and “I do things without thinking”) also ranked high for both genders, but the latter showed a slightly stronger effect for males. A history of *Gambling* was a shared risk factor across both groups, with a strong effect for males. Although *Gang Membership* was a differentiating indicator for both genders, it ranked third for males and showed a much stronger contribution to their model compared to females. *Justifies Hitting Back* also appeared as an important feature for both groups, with a slightly stronger influence for females.

On the protective side, *Does School Work* was a key feature for both genders, with a slightly stronger effect among males. *Parents Heard about Bad Behavior* appeared only in the top 10 features for females, whereas *Teachers Believe I can Do Well* was among the top indicators only for males. Overall, among the top 10 features in the classification of cannabis use, only one item differed across genders, along with a minor shift in the order of importance (Figs. 3C/D, 4B).

## Discussion

These findings underscore the centrality of school climate in differentiating youth substance users. In the current study, this set of school climate and related items demonstrated robust classification power for both cannabis and alcohol use. This result is consistent across different modeling choices (e.g., with or without SMOTE), suggesting that school climate is not merely an ancillary factor but rather a core component in understanding adolescent risk behaviors. The differences in classification accuracy between cannabis and alcohol users highlight the nuanced ways in which school climate indicators are associated with substance use. Specifically, the stronger classification capacity for cannabis compared to alcohol may point to the heightened role of psychosocial and relational dimensions—such as emotional regulation, teacher support, and school satisfaction—in shaping cannabis use. These findings align with prior research suggesting that individuals who struggle with emotional regulation or feel less connected to school environments may be at heightened risk for certain types of substance use (Cornell & Huang, 2016; Ko et al., 2023; Patte et al., 2017).

We identified 20 features from a broader list of school climate measurement scales (154 items) that exhibit strong classification accuracy for cannabis and alcohol use. These items reflect alignment with prior literature that demonstrates associations between supportive social relationships and lowered likelihood to use substances (Wormington et al., 2013 and externalizing behavior problems and alcohol and cannabis use (De Geronimo et al., 2024). Moreover, having 15 shared indicators suggests a convergence of underlying risk and protective processes for these two substances. This convergence is informative for practitioners and policymakers, as prevention and intervention strategies targeting common factors—such as absenteeism, behavioral challenges, and teacher support—may yield benefits across multiple substance types.

Although many features functioned similarly for males and females, the magnitude of their differentiating effects varied by substance. Notably, our findings indicate that cannabis use is particularly sensitive to protective factors among females, suggesting that bolstering school engagement and teacher support could have a disproportionately positive effect on reducing cannabis use in this subgroup. These results align with broader research on adolescent development, where gender-specific pathways often emerge, signaling the need for tailored prevention efforts. For instance, *Weapon Carrying* and *Gang Membership* remain strong risk factors for alcohol use only for males. These subtle differences in how aggression and emotional regulation relate to substance use may warrant gender-responsive interventions.

Although grounded in statistical learning theory (Bennett et al., 2022), our ML findings are consistent with well-established correlates identified in prevention science, such as age, school engagement, and behavioral risk indicators. However, our analytic approach offers several methodological advantages over traditional regression models. ML methods, particularly when paired with explainability tools like SHAP, enable the modeling of complex, nonlinear relationships and interactions without the need for prior specification. This allows researchers to uncover nuanced patterns within large, high-dimensional datasets that may remain undetected using traditional regression frameworks (Bzdok et al., 2018; Obermeyer & Emanuel, 2016). For example, identifying the top 10 predictors of substance use from over 150 candidate variables highlights the utility of ML in distilling intricate associations into actionable features for targeted prevention strategies and risk identification. Furthermore, explainability tools facilitate comparison of the relative importance and predictive strength of features across subgroups, such as gender, enhancing the precision of intervention design. Moreover, our use of cross-validation and classification metrics like precision and MCC ensures that the models generalize well to real-world data, particularly for identifying minority-class cases such as substance users—an area where logistic regression often underperforms (Christodoulou et al., 2019). Therefore, this study should be viewed not only as a confirmation of existing findings but also as an illustration of how interpretable ML can enrich prevention research by improving classification accuracy and model transparency.

The application of ML algorithms, particularly with techniques like SMOTE, facilitated the reliable classification of substance use and demonstrated the relative importance of different predictors. This underscores the utility of advanced analytical methods in parsing complex, multifaceted constructs, such as school climate. While SMOTE was used only during training and applied conservatively to minimize distortion, it may still introduce synthetic patterns that influence feature importance estimates to some extent. However, our sensitivity analyses indicated high consistency in feature rankings across SMOTE and non-SMOTE models, suggesting that the identified differentiators reflect meaningful patterns in real-world data rather than modeling artifacts. The observed variations in classification accuracy and feature importance across different modeling decisions also highlight the need for methodologically rigorous approaches. Researchers and practitioners should carefully select and fine-tune algorithms while considering class imbalance and variable selection, acknowledging that a portion of feature importance may be shaped by modeling artifacts, particularly when synthetic data are used. These considerations are essential to ensure robust and interpretable models. In addition, the application of SHAP analysis proves instrumental in identifying both universal and gender-specific predictors within the context of alcohol and cannabis use. The observed shifts in classification magnitude and ranking when stratified by gender emphasize the value of subgroup analyses in uncovering latent patterns that might otherwise remain obscured in aggregate-level models. These findings advocate for the integration of interpretable ML tools, such as SHAP, into behavioral and epidemiological research to advance the precision and inclusivity of data-driven insights.

### Limitations and Future Directions

Despite the robust classification performance observed, this study is subject to several limitations. First, the cross-sectional and self-report design limits our ability to draw causal inferences regarding the observed associations. Second, although the SMOTE was applied conservatively and did not substantially affect feature importance ranking, it still involves the creation of synthetic cases, which may introduce subtle artifacts. While our results are stable across models, caution is still warranted when interpreting predictors trained on oversampled data. Third, the ML algorithms employed here, while powerful, can sometimes obscure the interpretability of complex interactions among predictors—an inherent trade-off between model performance and transparency. Finally, the study’s sample may not be fully generalizable to other demographic (i.e., elementary) or regional populations, underscoring the need for replication and validation in diverse settings. Additionally, gender was represented only as male and female. Future research may benefit from extending this approach to include non-binary or gender-diverse youth and other factors, such as age, socioeconomic status, or ethnicity, to further disentangle the multifaceted dynamics of risk and resilience in adolescent substance use. Longitudinal studies would also allow for a more robust set of classification models. Additional research is needed to test, perhaps through intervention studies, the extent to which these core factors are associated with subsequent use of alcohol and cannabis.

## Conclusion

Taken together, these results illustrate that indicators of school climate play a potentially important role in differentiating youth cannabis and alcohol users, with notable commonalities and distinctions across substances and gender. By focusing on a core set of variables—many of which capture behavioral tendencies, emotional well-being, and perceptions of teacher and school support—interventions can be more effectively tailored to the specific dynamics that influence adolescent substance use. Future research might build on these findings by investigating additional contextual and developmental factors, as well as by validating the identified differentiators in diverse populations and across varying school settings.

## Supplementary Information

Below is the link to the electronic supplementary material.

ESM 1 (DOCX 1.07 MB)

ESM 2 (DOCX 26.0 KB)

## Data Availability

The middle school data are available at Comprehensive Assessment of School Climate to Improve Safety in Maryland Middle Schools, 2015-2018 (umich.edu) https://doi.org/10.3886/ICPSR37488.v1.
