# Supplementary material for: Leveraging Machine Learning to Understand the Link Between School Climate and Youth Substance Use: a Focus on Cannabis and Alcohol Use
Source: Prev Sci. 2026 Feb 28;27(7):1064–76. doi: 10.1007/s11121-026-01887-2 (PMC13550096; doi:10.1007/s11121-026-01887-2)
Supplement: Supplementary file 1 [file 11121_2026_1887_MOESM1_ESM.docx]

**Supplemental Table 1. Full List of Variables from the MDS3 School Climate Measure Included in the Machine Learning Analysis**

| **Variable Name** | **Description** |  |
| --- | --- | --- |
| U031D6St | Age (continuous) |  |
| ***Response Options: 1 = male or 2 = female*** | |  |
| U031D1 | Gender |  |
| ***Response Options: 0 = no or 1 = yes*** | |  |
| U031D3id_1 | Race: Native American/ American Indian |  |
| U031D3id_2 | Race: White |  |
| U031D3id_3 | Race: Hispanic/ Latino |  |
| U031D3id_4 | Race: Asian/ Pacific Islander |  |
| U031D3id_5 | Race: Black/ African American |  |
| U031D3id_6 | Race: Native Hawaiian or Other Pacific Islander |  |
| U031D3id_7 | Race: Other |  |
| ***Response Options: 1 = strongly disagree; 2 = disagree; 3 = agree; 4 = strongly agree*** | |  |
| U031SV5R | I feel safe going to and from this school.^a^ |  |
| U031SV6R | Students at this school try to stop bullying.^a^ |  |
| U031SV8R | Adults at this school try to stop bullying.^a^ |  |
| U031SE1R | My teachers make me feel good about myself.^a^ |  |
| U031SE2R | My teachers tell me when I do a good job.^a^ |  |
| U031SE4R | I enjoy learning at this school.^a^ |  |
| U031SE5R | I like coming to school.^a^ |  |
| U031SE6R | I do all my school work.^a^ |  |
| U031SE7R | I like this school.^a^ |  |
| U031SE8R | Teachers at this school do or say things that make students feel bad about themselves.^b^ |  |
| U031AE1R | My teachers believe that I can do well in school.^a^ |  |
| U031AE2R | I believe I can do well in school.^a^ |  |
| U031AE3R | My teachers encourage me to work hard in my classes.^a^ |  |
| U031AE4R | My teachers always want me to do my best.^a^ |  |
| U031AE5R | It is important to finish high school.^a^ |  |
| U031AE6R | Teachers believe all students can do well if they try.^a^ |  |
| U031MR1R | At school I do interesting activities.^a^ |  |
| U031MR2R | I help decide things like class activities or rules.^a^ |  |
| U031MR3R | At school I do things that make a difference.^a^ |  |
| U031FI1R | My parent(s) or guardian(s) feels welcome at this school.^a^ |  |
| U031FI2R | If I do something bad at school, my parent(s) or guardian(s) hears about it.^a^ |  |
| U031FI3R | When I do something good at school, my parent(s) or guardian(s) usually hears about it.^a^ |  |
| U031FI4R | The school tries to involve parents or guardians.^a^ |  |
| U031FI5R | Parents or guardians often come to my school to help out.^a^ |  |
| U031EI1R | It is okay to hit someone if they hit me first.^b^ |  |
| U031EI2R | If people do something that makes me really mad, they deserve to be beaten up.^b^ |  |
| U031EI3R | I believe that revenge is a good thing.^b^ |  |
| U031EI4R | I believe it is okay to hurt people who hurt you first.^b^ |  |
| U031CO1R | At this school, I feel like I belong.^a^ |  |
| U031CO2R | At this school, students like one another.^a^ |  |
| U031CO3R | At this school, I feel close to people.^a^ |  |
| U031CO4R | At this school, I feel like I am part of this school.^a^ |  |
| U031CO5R | At this school, my teachers notice when I am not there.^a^ |  |
| U031CO6R | At this school, my teachers listen to me when I have something to say.^a^ |  |
| U031CO7R | At this school, students help one another.^a^ |  |
| U031CO8R | At this school, my teachers care about me.^a^ |  |
| U031CO9R | At this school, students trust one another.^a^ |  |
| U031CO10R | At this school, students trust the teachers.^a^ |  |
| U031CO11R | At this school, students respect the teachers.^a^ |  |
| U031CO12R | At this school, students respect one another.^a^ |  |
| U031CO13R | At this school, teachers respect the students.^a^ |  |
| U031CO16R | At this school, the staff get along well.^a^ |  |
| U031CO20R | At this school, students and staff feel pride in this school.^a^ |  |
| U031OD1R | At this school, it is easy for teachers at my school to control the students.^a^ |  |
| U031OD2R | At this school, students disobey the rules.^b^ |  |
| U031OD3R | At this school, students listen to the teachers.^a^ |  |
| U031OD4R | At this school, it is usually very noisy.^b^ |  |
| U031OD5R | At this school, disruptions by other students can get in the way of my learning.^b^ |  |
| U031OD6R | At this school, teachers can handle students who disrupt class.^a^ |  |
| U031OD7R | At this school, there are clear rules about student behavior.^a^ |  |
| U031OD8R | At this school, misbehaving students get away with it.^b^ |  |
| U031OD9R | At this school, students get along well with each other.^a^ |  |
| U031OD10R | At this school, students are rewarded for positive behavior.^a^ |  |
| U031OD11R | At this school, everyone knows what the school rules are.^a^ |  |
| U031FC1R | At this school, students of all races are treated the same.^a^ |  |
| U031FC3R | At this school, all students are treated the same, regardless of whether their parents are rich or poor.^a^ |  |
| U031FC4R | At this school, boys and girls are treated equally well.^a^ |  |
| U031FC8R | The school provides instructional materials that reflect my culture, ethnicity, and identity.^a^ |  |
| U031SR1R | The school has programs to deal with violence and conflict between students.^a^ |  |
| U031SR2R | Teachers at my school help students with their problems.^a^ |  |
| U031SR4R | Students who need help for their problems are able to get it through school.^a^ |  |
| U031SR5R | There is someone at school who I can talk to about personal problems.^a^ |  |
| U031SR6R | Students have enough school supplies.^a^ |  |
| U031SR7R | Students have healthy food choices at school.^a^ |  |
| U031SR8R | The school provides adequate health services for students.^a^ |  |
| U031SR9R | Students have enough access to drinking water at school.^a^ |  |
| U031SR10R | There are lots of chances to get involved in sports, clubs, and other activities outside of class at this school.^a^ |  |
| U031PS1R | The principal at my school cares about us students.^a^ |  |
| U031PS5R | The school administration works closely with students to solve problems.^a^ |  |
| U031PD1R | There are often broken windows, doors, or desks in this school.^b^ |  |
| U031PD2R | The bathrooms in this school are clean.^a^ |  |
| U031PD3R | The school is usually clean and well-maintained.^a^ |  |
| U031PD4R | The temperature in this school is comfortable all year round.^a^ |  |
| U031PD5R | Vandalism of school property is a problem at this school.^b^ |  |
| U031PD6R | This school has a bright and pleasant appearance.^a^ |  |
| U031CR1R | Our class stays busy and doesn't waste time.a |  |
| U031CR2R | In this class, we learn a lot and stay busy almost every day.^a^ |  |
| U031CR3R | My teacher makes learning enjoyable.^a^ |  |
| U031CR4R | Student behavior in this class is under control.^a^ |  |
| U031SC1R | I feel like I can make a difference in my community.^a^ |  |
| U031SC2R | I feel like I belong in my community.^a^ |  |
| U031SC3R | I feel close to other people in my community.^a^ |  |
| U031WE29R | I have a strong sense of belonging to my own racial/ethnic group.^a^ |  |
| U031WE31R | I turn to my spiritual beliefs for when when I have personal problems or problems at school.^a^ |  |
| U031FA1R | I will go to college after I graduate.^a^ |  |
| U031FA2R | I am excited about my future.^a^ |  |
| U031FA3R | I have goals in my life.^a^ |  |
| U031FA4R | I can find lots of ways around any problem.^a^ |  |
| U031WE9R | I am physically fit.^a^ |  |
| U031WE13R | Get into a lot of trouble.^b^ |  |
| U031WE14R | Try to do what is right.^a^ |  |
| U031WE15R | Do well in school.^a^ |  |
| ***Response Options: 1 = not a problem; 2 = small problem; 3 = somewhat a problem; 4 = large problem*** | |  |
| U031SV9R | Physical fighting between students:^b^ |  |
| U031SV10R | Students carrying guns or knives:^b^ |  |
| U031SV12R | Harassment or bullying of students:^b^ |  |
| U031SV13R | Gang related activity:^b^ |  |
| U031WE24R | Students being hit, slapped, or kicked by their boyfriend or girlfriend:^b^ |  |
| U031WE25R | Students being threatened, degraded, or intimidated by their boyfriend or girlfriend:^b^ |  |
| ***Response Options: 1 = strongly agree; 2 = agree; 3 = disagree; 4 = strongly disagree*** | |  |
| U031AE7 | The students in this school do just enough to get by on their schoolwork.^a^ |  |
| ***Response Options: 1 = never; 2 = sometimes; 3 = often; 4 = almost always*** | |  |
| U031EI5R | I have trouble controlling my temper.^b^ |  |
| U031EI6R | I have threatened to hit or hurt someone.^b^ |  |
| U031EI7R | I do things without thinking.^b^ |  |
| U031EI8R | I get mad easily.^b^ |  |
| U031EI9R | I am lonely.^b^ |  |
| U031EI10R | I am sad.^b^ |  |
| U031EI11R | I am worried something bad is going to happen.^b^ |  |
| U031EI12R | I feel depressed.^b^ |  |
| U031EI13R | I feel nervous or anxious.^b^ |  |
| U031WE17R | Have trouble falling asleep.^b^ |  |
| U031WE18R | Feel you did not get enough sleep or rest.^b^ |  |
| U031WE19R | Feel stressed.^b^ |  |
| U031WE20R | Felt that difficulties were piling up so high that you could not overcome them.^b^ |  |
| ***Response Options: 1= not important at all; 2 = somewhat not important; 3 = somewhat important; 4 = very important*** | |  |
| U031WE30R | How important (if at all) is your faith to you?^a^ |  |
| ***Response Set: 1 = excellent; 2 = very good; 3 = good; 4 = fair; 5 = poor*** | |  |
| U031WE1 | Would you say that in general your health is:^b^ |  |
| ***Response Options: 0 = none; 1 = once; 2 = twice; 3 = 3 times; 4 = 4 times; 5 = 5 or more times*** | |  |
| U031WE3 | During the past 24 hours (yesterday), how many times did you drink soda pop:^b^ |  |
| U031WE4 | During the past 24 hours (yesterday), how many times did you eat fruit (Do not count fruit juice):^a^ |  |
| U031WE5 | During the past 24 hours (yesterday), how many times did you eat vegetables (Include salads and non-fried potatoes):^a^ |  |
| ***Response Options: 0 = no or 1 = yes*** | |  |
| U031WE10R_0_1 | During the past 12 months, have you had an episode of asthma or an asthma attack?^b^ |  |
| U031WE11R_0_1 | Have you ever used the Health Center at your current school for information or services? ^b^ |  |
| U031WE21R_0_1 | Are you currently in a romantic or dating relationship? |  |
| U031WE26_1 | Have you ever bet on the lotteries or bought a lottery ticket?^b^ |  |
| U031WE26_2 | Have you ever played cards or dice for money?^b^ |  |
| U031WE26_3 | Have you ever bet on horses or sports?^b^ |  |
| U031WE26_4 | Have you ever gambled in a casino?^b^ |  |
| U031WE26_5 | Have you ever played slot or poker machines?^b^ |  |
| U031WE26_6 | Have you ever gambled online?^b^ |  |
| U031WE26_8 | I have never gambled.^a^ |  |
| U031WE26_7 | Have you ever participated in another form of gambling?^b^ |  |
| U031SV24R | Have you ever belonged to a gang?^b^ |  |
| U031BU1R_0_1 | During the current school year have you been bullied?^b^ |  |
| U031CB2R_binary | In the past 3 months, have you been “cyberbullied?”^b^ |  |
| U031BU5R_0_1 | In the past 30 days, have you seen someone else being bullied?^b^ |  |
| ***Response Options: 1 = not at all; 2 = 1 time during the month; 3 = 2-3 times during the month; 4 = once a week or several times a week*** | |  |
| U031BU3R_0_4 | During the past 30 days, how often have you been bullied?^b^ |  |
| U031BU4R_0_4 | In the past 30 days, how often have you bullied someone else?^b^ |  |
| ***Response Options: 1 = yes or 2 = no*** | |  |
| U031BU13R | This school year I have been bullied by teachers or staff.^a^ |  |
| U031BU19 | During the current school year, have you experienced personal harassment or bullying on school property?^a^ |  |
| ***Response Options: 0 = 0 days; 1 = 1 day; 2 = 2 or 3 days; 3 = 4 or 5 days; 4 = 6 or more days*** | |  |
| U031SV19R | During the past 30 days, how often did you carry a weapon, such as a knife or gun, on school property?^b^ |  |
| U031SV21R | During the past 30 days, how many days did you not go to school because you felt you would be unsafe at school or going to and from school?^b^ |  |
| ***Response Options: 0= 0 times; 1= 1 time; 2 = 2 or 3 times; 3 = 4 or 5 times; 4 = 6 or 7 times; 5 = 8 or 9 times; 6 = 10 or 11 times; 7 = 12 or more times*** | |  |
| U031SV20R | During the past 12 months, how many times were you in a physical fight on school property?^b^ |  |
| U031SV22R | During the past 12 months, how many times were you threatened or injured with a weapon on school property?^b^ |  |
| ***Response Options: 1= very underweight; 2 = slightly underweight; 3 = about the right weight; 4 = slightly overweight; 5 = very overweight*** | |  |
| U031WE6 | How do you describe your weight? |  |
| ***Response Options: 1= lose weight; 2 = gain weight; 3 = stay the same weight; 4= I am not trying to do anything about my weight*** | |  |
| U031WE7 | Which of the following are you trying to do about your weight? |  |
| ***Response Options: 0= 0 days; 1 = 1 day; 2= 2 days; 3 = 3 days; 4 = 4 days; 5 = 5 days; 6 = 6 days; 7= 7 days*** | |  |
| U031WE8 | On how many of the past 7 days did you exercise or do a physical activity for at least 20 minutes that made you sweat or breathe hard?^a^ |  |
| ***Response Options: 1 = none; 2 = 1 say; 3 = 2-3 days; 4 = 4 or more days*** | |  |
| U031SV23R2 | During the last month, how many days of school have you missed because you skipped or "cut"?^b^ |  |
| ***Response Options: 1= walk/bike/skateboard; 2 = ride the bus; 3 = ride in a car driven by a parent; 4 = ride in a car driven by another student; 5 = drive yourself*** | |  |
| U031SV26 | How do you usually get to and from school? | |
| ^a^higher scores indicate a more favorable outcome  ^b^lower scores indicate a more favorable outcome | | |

| **Supplemental Table 2: Recursive Feature Elimination Results** | | | | | | | |  |
| --- | --- | --- | --- | --- | --- | --- | --- | --- |
|  | **Model** | **Accuracy** | **Precision** | **Recall** | **F1 Score** | **Cohen Kappa** | **Matthews  Corrcoef** | **ROC AUC** |
| **Alcohol** |  | 0.8942 | 0.8983 | 0.8942 | 0.894 | 0.7886 | 0.7926 | 0.8944 |
|  | XGBoost |  |  |  |  |  |  |  |
| **Cannabis** |  |  |  |  |  |  |  |  |
|  | Extra Tree | 0.9339 | 0.9364 | 0.9339 | 0.9338 | 0.8679 | 0.8704 | 0.9341 |

MCC: **Matthews Correlation Coefficient**

ROC- AUC: Receiver Operating Characteristic - Area Under the Curve

| **Supplemental Table 3: Classification Model Results** | | | | | | | | | |  |
| --- | --- | --- | --- | --- | --- | --- | --- | --- | --- | --- |
| **A - Cannabis with smote** | | | |  |  |  |  |  |  |  |
| **Model** | **Accuracy** | **Precision** | | **Recall** | **F1 Score** | **Cohen Kappa** | **Matthews Corrcoef** | **ROC  AUC** |  |  |
| Extra Trees | 0.88 | 0.86 | | 0.88 | 0.86 | 0.31 | 0.35 | 0.85 |  |  |
| Random Forest | 0.88 | 0.86 | | 0.88 | 0.87 | 0.34 | 0.37 | 0.85 |  |  |
| XGboost | 0.86 | 0.87 | | 0.86 | 0.87 | 0.42 | 0.42 | 0.86 |  |  |
| GBC | 0.89 | 0.87 | | 0.89 | 0.87 | 0.37 | 0.39 | 0.86 |  |  |
|  |  |  | |  |  |  |  |  |  |  |
| **B- Cannabis without Smote** | | | |  |  |  |  |  |  |  |
| **Model** | **Accuracy** | **Precision** | | **Recall** | **F1 Score** | **Cohen Kappa** | **Matthews Corrcoef** | **ROC  AUC** |  |  |
| Extra Trees | 0.79 | 0.88 | | 0.79 | 0.82 | 0.37 | 0.42 | 0.86 |  |  |
| Random Forest | 0.86 | 0.87 | | 0.86 | 0.86 | 0.42 | 0.42 | 0.86 |  |  |
| XGBoost | 0.77 | 0.88 | | 0.77 | 0.81 | 0.36 | 0.41 | 0.87 |  |  |
| GBC | 0.89 | 0.87 | | 0.89 | 0.87 | 0.36 | 0.39 | 0.87 |  |  |
|  |  |  | |  |  |  |  |  |  |  |
| **C- Alcohol with Smote** | | |  |  |  |  |  |  |  |  |
| **Model** | **Accuracy** | **Precision** | | **Recall** | **F1 Score** | **Cohen Kappa** | **Matthews Corrcoef** | **ROC  AUC** |  |  |
| Extra Trees | 0.82 | 0.80 | | 0.82 | 0.81 | 0.38 | 0.39 | 0.82 |  |  |
| Random Forest | 0.82 | 0.81 | | 0.82 | 0.81 | 0.40 | 0.40 | 0.83 |  |  |
| XGBoost | 0.79 | 0.82 | | 0.79 | 0.80 | 0.42 | 0.43 | 0.84 |  |  |
| GBC | 0.83 | 0.81 | | 0.83 | 0.81 | 0.37 | 0.39 | 0.84 |  |  |
|  |  |  | |  |  |  |  |  |  |  |
| **D- Alcohol without Smote** | | | |  |  |  |  |  |  |  |
| **Model** | **Accuracy** | **Precision** | | **Recall** | **F1 Score** | **Cohen Kappa** | **Matthews Corrcoef** | **ROC  AUC** |  |  |
| Extra Trees | 0.75 | 0.82 | | 0.75 | 0.77 | 0.39 | 0.42 | 0.84 |  |  |
| Random Forest | 0.75 | 0.83 | | 0.75 | 0.77 | 0.40 | 0.43 | 0.84 |  |  |
| XGBoost | 0.74 | 0.83 | | 0.74 | 0.77 | 0.39 | 0.43 | 0.85 |  |  |
| GBC | 0.83 | 0.82 | | 0.83 | 0.82 | 0.39 | 0.41 | 0.85 |  |  |

Figures

**Supplemental Figure 1:** Top 10 Feature Importance for Cannabis and Alcohol Use without SMOTE application (XGBoost Model)

*Note:* SHAP summary plots for alcohol use (A) and cannabis use (B) show feature importance and the direction of influence on model features. Each point represents an individual data instance, with color indicating the feature value (red = high, blue = low).

**Supplemental Figure 2:** Top Feature Importance for Cannabis and Alcohol Use for Overall Model Results

 *Note:* SHAP summary plots for alcohol use (A) and cannabis use (B) highlight the feature importance and direction of influence on the model classification (class 1 = substance use). Each point represents an individual data instance, with color indicating the feature value (red = high, blue = low).

**Supplemental Figure 3:** Top 10 Feature Importance for Cannabis and Alcohol Use across Gender

 *Note:* SHAP summary plots for alcohol use (A) and cannabis use (B) highlight the feature importance and direction of influence on the model classification (class 1 = substance use). Each point represents an individual data instance, with color indicating the feature value (red = high, blue = low).
